# Strategies for screening, occupational prevention, and management of COVID-19 in outpatient clinics in Shandong

**DOI:** 10.3389/fpubh.2022.911364

**Published:** 2022-09-15

**Authors:** Yan Zhang, Yan Lu, Juan Tang, Yu Sun, Ze-Hua Zhao, Xiang-Dong Jian, Xi-Mei Gao

**Affiliations:** ^1^Department of International Medicine, Qilu Hospital of Shandong University, Jinan, China; ^2^Department of Nursing, Qilu Hospital of Shandong University, Jinan, China; ^3^Department of Hepatology, Qilu Hospital of Shandong University, Jinan, China; ^4^Department of Poisoning and Occupational Diseases, Emergency Medicine, Qilu Hospital of Shandong University, Jinan, China

**Keywords:** COVID-19, occupational exposure, outpatient clinic, prevention, epidemiological investigations

## Abstract

**Objective:**

We hope to analyze the information of outpatients in a tertiary care hospital during the epidemic of COVID-19, so as to formulate effective regulations for the prevention and control of COVID-19.

**Methods:**

We collected information from outpatients from January 28, 2020 to March 2, 2020 and performed the statistical analysis.

**Results:**

During the study period, there were more than 60,000 outpatients. Among them, 404 patients with a body temperature above 37.3°C who had not been to Wuhan and had no contact with people from Wuhan. There were 8 people who had contact with people from Wuhan, such as 4 people with fever, 3 people with normal body temperature but cough symptoms, and 1 person with normal body temperature and no other discomfort. There were 2 patients with high body temperature from the epidemic area in Wuhan, and one novel Coronavirus patient was confirmed as the final result.

**Conclusion:**

During the COVID-19 pandemic, outpatient medical staff should enhance their awareness of protection, hospitals should standardize the outpatient COVID-19 prevention and control system, improve the prevention and emergency system, and reduce occupational exposure hazards and the occurrence of post-exposure infections.

## Introduction

Since December 2019, pneumonia cases caused by an unknown pathogen have been observed in Wuhan, Hubei. The epidemiological investigations have revealed high contagiousness and potential of transmission among people. In January 2020, the pathogen was isolated and identified by scientists. Genome analysis showed that the pathogen was a novel coronavirus and named COVID-19 (1). The COVID-19 pandemic suggests the importance of infection prevention and control measures in health facilities (2). The rigorous measure has been taken to prevent and control the spread of the COVID-19 in China. As the cases of COVID-19 have been reported nationwide, the outpatient clinics of hospitals in other provinces apart from Hubei are also exposed to the infected patients. Without sufficient awareness of occupational protection and appropriate regulation of outpatient activity, it is highly possible that nosocomial spread occurs (3). It has been required that infectious diseases be managed by different classifications and the healthcare providers are obliged to prevent, control, and eliminate the spread of infectious diseases according to the Infectious Disease Prevention Act in China (4). It is acknowledged that the outpatient clinics are the first guard to the potential virus infection. And due to the fact that long duration and great workload are common for medical staff in outpatient clinics, the possibility of occupational exposure for them is greatly increased. To propose better working protocols and strategies for the occupational prevention of COVID-19, we summarized the characteristics of the outpatients and testified the routine preventive measures.

## Materials and methods

### Subjects

From January 28, 2020 to March 2, 2020, all patients who attended the outpatient clinic of the Qilu Hospital of Shandong University were included. Prior to the information collection, informed consent was obtained from each participant and the study was approved by the local Ethical Committee of Qilu Hospital of Shandong University.

### Epidemiological analysis

We detected body temperature and developed a questionnaire to collect relevant information on outpatients during the study period, such as the motion trail in hospital, history of travel to epidemic areas, and potential exposure to suspected patients. All registered information was entered into an Excel sheet and sorted and summarized.

## Results

### The daily outpatient amount

**Figure 1:**
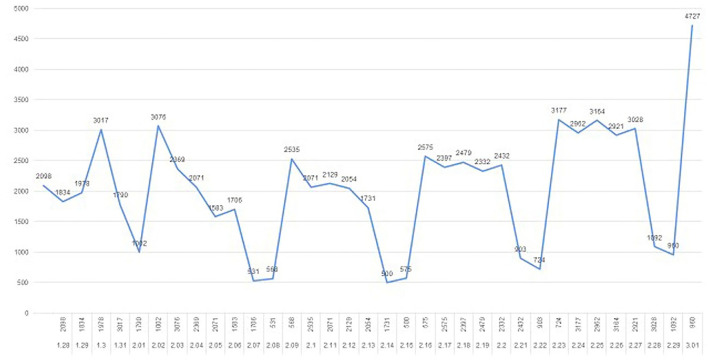
The daily amount of outpatient visit.

The total amount of outpatient visits from January 28, 2020 to March 1, 2020 was over 60, 000 (Figure 1). The maximum amount of daily outpatient visits was 4,727 on March 1, 2020. The daily outpatient amount was fluctuated and relatively low during the weekends. At the end of February, the daily outpatient amount was increased compared to the former part of the month.

### Epidemiological analysis of outpatients with high risk of COVID-19

All outpatients were subjected to epidemiological investigations. A total of 404 patients had a body temperature above 37.3°C without a history of traveling to Wuhan or coming from the epidemic area of Wuhan (Table 1). A total of 8 patients had a history of traveling to Wuhan, of which 4 patients had a fever and 3 patients had a symptom of cough with a normal body temperature. A total of 2 patients came from the epidemic area of Wuhan with an abnormal temperature, of which one was diagnosed with COVID-19 (Table 2).

**Table 1:** Fevered patients without history of coming from the epidemic area of Wuhan.

**Department**	**Number**	**Direction**
Department of pediatrics	274	Fever clinic
Department of internal medicine	85	Fever clinic
Department of obstetrics and gynecology	13	Fever clinic
Department of clinical laboratory	9	Fever clinic
Department of otorhinolaryngology	5	Fever clinic
Department of oncology	5	Fever clinic
Department of ultrasound	5	Fever clinic
Department of productive medicine	4	Fever clinic
Department of International medicine	2	Fever clinic
Department of hepatology	1	Fever clinic
Department of surgery	1	Fever clinic

**Table 2:** Patients with a history of traveling to Wuhan or coming from the epidemic area of Wuhan.

**Department**	**Number**	**Symptom**	**Epidemiological background**	**Direction**
Department of internal medicine	1	Body temperature above 37.3°C	From the epidemic area of Wuhan	Fever clinic, designated hospital (diagnosed with COVID-19)
Computerized tomography room	1	Body temperature above 37.3°C	From the epidemic area of Wuhan	Fever clinic, designated hospital
Department of pediatrics	7	4 had fever, 3 had cough	Travel to Wuhan	Fever clinic
Bronchoscope room	1	None	Travel to Wuhan	Fever clinic

## Prevention and management strategies

### Strengthen the management of personnel

The patients and their companions are the major sources of the floating population at outpatient clinics. To help prepare the patients for the appointment and seeing the doctors, the visit procedure and notes were broadcasted by the Internet, WeChat, and electronic screens. These measures can shorten the stay time of patients and their companions in the department of outpatient. The patients and their companions are required to wear surgical masks or N95 masks and keep a distance of at least 1 meter from each other all along their visiting time. Before they enter the hospital, the patients and their companions should receive body temperature detection and show their ID cards. And epidemiological investigations help to learn about the purpose of the visit, history of travel to Hubei, potential exposure to COVID-19 diagnosed or suspected patients, and possible contact with clustering infection households.

The medical staffs are crucial to take part in the prevention of nosocomial COVID-19. The doctors and nurses should detect and report their body temperature every day. Once they had a fever or symptoms, such as cough or chest distress, they should not come to work. The working staff should obey the standard prevention rules, evaluate the potential risk of occupational exposure when they operate and pay much attention to hand hygiene (5). Furthermore, the working stall at outpatient clinics should be equipped with personal protection facilities, such as isolation gowns, medical hats surgical masks, latex gloves, protection suits, goggles, and face shields, if necessary. A three-level previewing and triaging system is applied and when the outpatients and their companions register and take the body temperature test, the doctors and nurses should keep a safe distance from them and use sanitizers to prevent contact infection. On the other hand, the amount of working staff can be adjusted according to the number of outpatient visits. Exquisite management of working shifts helps to ensure sufficient rest of healthcare providers and decreases the unnecessary consumption of protection facilities. A team for an emergency situation can be set up to cope with the unexpected inflow of large amounts of patients.

Apart from the doctors and nurses, other working staff, such as cleaners and security personnel, should also be trained for nosocomial infection prevention and personal protection. The cleaners should disinfect the working environment using chlorine-containing disinfectant twice daily. And the medical waste should be transported and disposed timely. Security patrols should be strengthened to maintain normal medical order and deal with emergency events.

### Enhance the management of processing

A three-level defense system is applied to prevent the nosocomial COVID-19 spread. The first line of defense is the previewing and triaging station in the emergency room, the hall of the outpatient department, and the entrances of the hospital. Medical staff should guide the patients and their companions to sign the consent, check the identification, detect and record the body temperature and investigate the epidemiological background. If the patient has a fever or exposure history, he should be required to wear a medical mask appropriately and be guided to the fever clinic on the assigned route. And the environment should be disinfected at once. The second line of defense is the previewing and triaging station at different departments. The nurses should pay attention to personal protection and hand hygiene. They should recheck the identification and epidemiological information of the patients and retest their body temperature. Patients with a temperature above 37.3°C should also be guided to fever clinic in the assigned route. The third line of defense is the doctors in the clinic rooms. The doctors at the department of outpatient should strengthen their personal protection and obey the rules of hand hygiene. They should detect the body temperature of the patient and inquire about the epidemiological information once again. And patients with high risk should be guided to the fever clinic by the nurses. More importantly, the process of the outpatient visit should obey the principle of unchangeableness, i.e., the requirement that only one patient can stay in the clinic room, the route of the outpatient visit, the place of patients registering, taking examinations, and getting the drugs, and the accompanying medical staff and companions should not be changed to avoid extra contact between patients and other people.

The nurses at the outpatient clinic can be subdivided into triage, service, and contact post. And flexible shifts are recommended. When the triage work is in need, nurses in triage posts should be sufficient to finish measuring body temperature, helping the patients get registered, and triaging quickly. After triaging, the nurses in the service post should guide the patients and their companions to take seats in a safe distance and wait for seeing the doctor. They should also monitor the patients and persuade them from close chatting. And the nurse in contact post should guide the patients to the clinic room, keep them in quiet, and in order, and make sure that only one patient can stay in the clinic room.

To better learn the situation, statistics on the fevered patients and their companions should be collected and analyzed daily. Also, the medical staff should report their exposure to the suspected patients to discover the unit with high risk and make corresponding responses. Senior nurses should survey and supervise the implementation of preventive measures.

### Improve the management of environment

The accommodation capacity of the patients and their companions are evaluated based on the available space and facilities at the department of outpatient. And the amount of outpatients is accordingly limited to avoid personnel overload and increased risk of COVID-19 spread. The ventilation of the clinic area is ensured by opening the window twice daily for at least 30 min. The air is refreshed using an air purifier with a circulating fan when the clinic room is used. And the air is disinfected utilizing an ultraviolet radiator or peroxyacetic acid and chlorine-containing disinfectant spray. The air-conditioning system and exhaust fans are regularly checked to function well. The air conditioner filter is cleaned and the air outlets are disinfected regularly. The public areas such as nurse stations, the hall, corridors, waiting areas, clinic rooms, and toilets are disinfected twice daily using chlorine-containing disinfectant twice daily. The medical and non-medical waste is cleared timely and the dustbins are disinfected with 75% ethanol or chlorine-containing disinfectant.

### Reinforce the management of emergency response

The three-level previewing and triaging system should be strictly executed. Once the patient with high risk who has fever or epidemiological hazards is identified, the patient should be immediately registered and reported. Specific staff in response to the emergencies is responsible for the patient transferring to the fever clinic. The space and materials which are exposed to the suspected patients should be disinfected sufficiently with chlorine-containing disinfectant or ultraviolet radiation.

## Case

A 37-year-old male traveled to Chongqing in business on 17th, January and flew back on 20th, January. He had a fever during this time and saw a doctor in a community hospital. His symptoms were worsened on 25th, January and came to the department of outpatient, Qilu Hospital of Shandong University. The nurses at the first level previewing and triaging station collected his epidemiological information and detected his body temperature. The emergency response was initiated after the nurses judged that this patient is at high risk of COVID-19. The personal information of this patient was registered and reported. The patient was transferred to the fever clinic for further examination. In this process, the two nurses were in protection suits from the beginning and strictly cleaned their hands and changed their suits according to the protocols. At the same time, the environment exposed to the patient was carefully disinfected. On 1st, February, the nucleic acid test of nasopharyngeal swab samples showed positive results, and the patient was diagnosed with COVID-19 after the expert consultation. The patent was then transferred to the designated hospital by ambulance. The two nurses were quarantined at home for 2 weeks and received medical observations. No discomforts or abnormal body temperatures were reported before the quarantine was relieved.

## Discussion

COVID-19 is an infectious disease that is managed according to the national regulations in China. Outpatient clinics are the first to be affected and are crucial in the prevention and control of nosocomial spread. The systems of information registration, screening process, visiting management, and emergency response play important roles in the management of outpatients and help to build defense lines in the prevention of COVID-19 transmission at the department of outpatient. First of all, awareness of occupational protection should be emphasized to avoid epidemic among the medical staff. Furthermore, the department of outpatient is responsible for the identification of potentially infected patients quickly and accurately (6). Thus, working protocols and management regulations are needed (7). Trainings are necessary and the information should be updated timely. Moreover, measures to isolate and monitor the fevered and suspected patients are important to protect the medical staff and other patients. Meanwhile, sufficient preparation and rigorous execution of the management regulations are vital to make sure the situation is under control.

In this study, the department of outpatient admitted more than 60,000 patients in this period. The 2 suspected patients were identified and transferred to the designated hospitals and 1 patient was diagnosed with COVID-19 eventually. Owing to the strict measures, standardized regulations and great execution of the protocols in the daily working at the department of outpatient, none of the medical staff was found to be infected. Therefore, improving the strategies for screening, occupational prevention, and management is vital and effective in the control of the COVID-19 epidemic at the department of outpatient.

## Data availability statement

The original contributions presented in the study are included in the article/supplementary material, further inquiries can be directed to the corresponding authors.

## Ethics statement

Ethical review and approval was not required for the study on human participants in accordance with the local legislation and institutional requirements. Written informed consent for participation was not required for this study in accordance with the national legislation and the institutional requirements.

## Author contributions

YZ and X-MG: methodology, formal analysis, data curation, software, writing—original draft, and visualization. YL and YS: writing—review and editing and project administration. YL, JT, and Z-HZ: investigation. X-DJ, YZ, and X-MG: conceptualization, resources, supervision, and project administration. All authors contributed to the article and approved the submitted version.

## Conflict of interest

The authors declare that the research was conducted in the absence of any commercial or financial relationships that could be construed as a potential conflict of interest.

## Publisher's note

All claims expressed in this article are solely those of the authors and do not necessarily represent those of their affiliated organizations, or those of the publisher, the editors and the reviewers. Any product that may be evaluated in this article, or claim that may be made by its manufacturer, is not guaranteed or endorsed by the publisher.
